# Phenolic Profile of Nipa Palm Vinegar and Evaluation of Its Antilipidemic Activities

**DOI:** 10.1155/2020/6769726

**Published:** 2020-09-04

**Authors:** Moragot Chatatikun, Wiyada Kwanhian

**Affiliations:** ^1^Department of Medical Technology, School of Allied Health Sciences, Walailak University, Nakhon Si Thammarat 80161, Thailand; ^2^Center of Excellence Research for Melioidosis (CERM), Walailak University, Nakhon Si Thammarat 80161, Thailand; ^3^Research Excellence Center for Innovation and Health Product, Walailak University, Nakhon Si Thammarat 80161, Thailand

## Abstract

Obesity and overweight are strongly associated with dyslipidemia which can promote the development of cardiovascular diseases. Recently, natural products have been suggested as alternative compounds for antioxidant and antilipidemic activity. The objective of this study was to determine the phenolic compounds and assess the inhibitory activities on pancreatic lipase, cholesterol esterase, and cholesterol micellization of nipa palm vinegar (NPV). Total phenolic content was assessed and phenolic compounds were determined using the Folin–Ciocalteu assay and liquid chromatography-mass spectrometry (LC-MS), respectively. Pancreatic lipase and cholesterol esterase inhibitory activities of the NPV were measured using enzymatic colorimetric assays. The formation of cholesterol micelles was assessed using a cholesterol assay kit. The phenolic content of NPV was 167.10 ± 10.15 *µ*g GAE/mL, and LC-MS analyses indicated the presence of gallic acid, isoquercetin, quercetin, catechin, and rutin as bioactive compounds. Additionally, the NPV inhibited pancreatic lipase and cholesterol esterase activities in a concentration-dependent manner. Moreover, the NPV also suppressed the formation of cholesterol micellization. These results suggest that phenolic compounds, especially gallic acid, isoquercetin, quercetin, catechin, and rutin, from NPV may be the main active compounds with possible cholesterol-lowering effects through inhibition of pancreatic lipase and cholesterol esterase activities as well as the inhibition of solubility of cholesterol micelles. Therefore, NPV may delay postprandial dyslipidemia, and it could be used as a natural source of bioactive compounds with antilipidemic activity. However, NPV should be extensively evaluated by animal and clinical human studies.

## 1. Introduction

Lipid metabolism disorders are commonly found in people who are obese. The prevalence of obesity and overweight has dramatically increased in developed and developing countries due to the increased consumption of high-fat diets and the daily intake of alcohol [1]. Obesity and overweight are associated with hyperglycemia and dyslipidemia in children and adolescents [2]. Dyslipidemia is a group of metabolic disorders and a noncommunicable disease manifested by elevation of serum cholesterol, low-density lipoprotein (LDL) cholesterol, and triglyceride concentrations and a decrease in high-density lipoprotein (HDL) cholesterol concentration. It is one of the major risk factors associated with atherosclerosis which leads to the development of cardiovascular diseases and increased mortality [3]. Orlistat is a weight loss agent which inhibits gastric and pancreatic lipases in the lumen of the gastrointestinal tract delaying absorption of dietary fat that is approved by the Food and Drug Administration for the treatment of obesity. It also improves total cholesterol and low-density lipoprotein for the treatment of dyslipidemia. The major side effects, which occur at an early stage of treatment with orlistat, are mainly gastrointestinal [4]. Simvastatin is a HMG-CoA reductase inhibitor which is commonly used to decrease blood cholesterol and triglyceride levels. The major adverse effects of statins are myositis, myalgia [5], rhabdomyolysis [6], and hepatic disorders [7].

Recently, natural products have been reported to have the potential to be developed faster and cheaper than conventional single drug. For example, curcumin, lycopene, monascin, ankaflavin, oleanolic acid, ursolic acid, berberine, amphene, tanshinone IIA, hesperetin, and naringenin inhibit cholesterol absorption in enterocytes [8]. Thus, natural products, especially phenolic compounds of these products, may inhibit pancreatic lipase, cholesterol esterase, and solubility of cholesterol micelles. Vinegar is generally used as a food condiment and as an alternative medicine for obesity [9], hyperlipidemia [10], hyperglycemia [11], and cancer [12] and as a disinfectant [13]. The nipa palm (*Nypa fruticans* Wurmb) vinegar has been used by Southeast Asia, and it has showed biological activities, such as antioxidant activity [14], antidiabetic activity [15], and hepatoprotective effects [16]. Therefore, the NPV could be a natural alternative to treat common diseases from contemporary diet. Nevertheless, the antilipidemic activity of this vinegar has not been previously evaluated. This study aimed to investigate phenolic compounds and *in vitro* inhibitory effects on pancreatic lipase, cholesterol esterase, and cholesterol micellization of nipa palm vinegar.

## 2. Materials and Methods

### 2.1. Chemicals and Reagents

All chemicals and reagents were of analytical grade. Folin–Ciocalteu phenol reagent, sodium carbonate, gallic acid, porcine pancreatic lipase, 4-methylumbelliferone, phosphate buffer saline, sodium citrate, orlistat, taurocholic acid, p-nitrophenylbutyrate (p-NPB), sodium phosphate buffer, sodium chloride, porcine pancreatic cholesterol esterase, simvastatin, cholesterol, oleic acid, phosphatidylcholine, methanol, and taurocholate salt were purchased from Sigma-Aldrich (St. Louis, MO).

### 2.2. Preparation of Nipa Palm Vinegar

Nipa palm sap was collected from Pak Phanang District, Nakhon Si Thammarat, Thailand (8°12′25.1″ N, 100°14′51.7″ E). All parts of the nipa palm were authenticated and a voucher number (*Nypa fruticans* Wurmb, voucher no. 01518) was deposited at Botanic Garden, Walailak University, Nakhon Si Thammarat, Thailand. The nipa palm sap was collected from cut stalks. The nipa palm sap was fermented to nipa palm vinegar by the local traditional method. In brief, the collected nipa palm sap was placed in terracotta jars for 40 days at room temperature to allow the natural fermentation process to occur. The level of acidity of nipa palm vinegar reaches 4 to 5%.

### 2.3. Determination of Total Phenolic Content

The total phenolic content was determined by the Folin–Ciocalteu assay as previously described [17]. In brief, 20 *µ*L of each sample was mixed with 100 *µ*L of Folin–Ciocalteu phenol reagent and 80 *µ*l of sodium carbonate solution (75 g/L). After incubation for 30 min at room temperature, the absorbance was measured at 765 nm. A calibration curve was plotted using gallic acid solutions (31.25, 62.5, 125, 250, and 500 *µ*g/mL). Total phenolic content was determined as *µ*g gallic acid equivalent per ml of nipa palm vinegar (*µ*g GAE/mL).

### 2.4. Determination of Phenolic Compounds by LC-MS Analysis

Nipa palm vinegar was subjected to commercial LC-MS analysis by the Central Laboratory Co., Ltd. (Bangkok, Thailand) essentially as described elsewhere [18]. Mass spectra data were recorded in ionization mode for a mass range of m/z 100–700. Phenolic standards from Sigma-Aldrich (St. Louis, MO) were gallic acid (≥99% purity), tannic acid (≥99% purity) and hydroquinone (≥99% purity), catechin (≥98% purity), rutin (≥94% purity), isoquercetin (98% purity), eriodictyol (98% purity), quercetin (95% purity), apigenin (≥95% purity), and kaempferol (≥97% purity), and their purity was determined by high-performance liquid chromatography (HPLC).

### 2.5. Pancreatic Lipase Inhibition Assay

Pancreatic lipase inhibition assay was undertaken as described by Adisakwattana et al. [19]. In brief, 25 *µ*L of nipa palm vinegar diluted with distilled water, or positive control (orlistat), was mixed with 25 *µ*L of porcine pancreatic lipase solution and 50 *µ*L of oleate ester of 0.1 mM fluorescent 4-methylumbelliferone (4-MUO) solution in phosphate buffer saline, subsequently. The mixture was incubated at 37^o^C for 20 min. Reaction was stopped by adding 100 *µ*L of 0.1 M sodium citrate at pH 4.2. The fluorescence of 4-methylumbelliferone released by the lipase was measured at excitation and emission wavelengths of 320 and 450 nm, respectively. Control without sample or orlistat represented 100% pancreatic lipase (PL) activity. The tests were performed in triplicate.

### 2.6. Pancreatic Cholesterol Esterase Inhibition Assay

The pancreatic cholesterol esterase inhibition assay was performed according to a previously published method [20]. In brief, different concentrations of nipa palm vinegar were incubated with mixtures containing 5.16 mM taurocholic acid, 0.2 mM p-nitrophenylbutyrate (p-NPB) in 100 mM sodium phosphate buffer, and 100 mM NaCl at pH 7.0. Porcine pancreatic cholesterol esterase at concentration of 1 *µ*g/mL was added into the reaction tube, and samples were incubated at 25°C for 5 min. After that, the absorbance of the solutions was measured at 450 nm. Simvastatin served as a positive control. Results were based on triplicate analysis.

### 2.7. Cholesterol Micellization Assay

Cholesterol micelles served as a model system for *in vitro* cholesterol micellization and were prepared according to a previous method [21]. In brief, the micelle solution (2 mM cholesterol, 1 mM oleic acid, and 2.4 mM phosphatidylcholine) was dissolved in methanol and then dried under nitrogen. After that, 15 mM phosphate-buffered saline (PBS) solution containing 6.6 mM taurocholate salt, pH 7.4, was added onto the dried micelles. The emulsion was sonicated twice for 30 min using a sonicator. The micelle solution was incubated overnight at 37°C. Various concentrations of nipa palm vinegar or equivalent PBS as a control were added into the micelle solutions, and samples were incubated for 2 h at 37°C. This mixture was then centrifuged at 16,000 rpm for 20 min. The supernatant of the mixture was collected for determination of cholesterol using total cholesterol test kits (Human Diagnostics Worldwide, Weisbaden, Germany). Gallic acid served as a positive control. All tests were taken in triplicate analysis.

### 2.8. Data Analysis

All analyses were carried out in triplicate, and data are expressed as mean ± standard deviation. The correlation coefficient (*R*^2^) was evaluated by using SigmaPlot version 12.2 software.

## 3. Results

### 3.1. Determination of Total Phenolic Content

The amount of total phenolics in nipa palm vinegar (NPV) was determined using the Folin–Ciocalteu method. The value was determined as *µ*g gallic acid equivalent per ml of NPV (*µ*g GAE/mL). The phenolic content of NPV was determined to be 167.10 ± 10.15 *µ*g GAE/mL.

### 3.2. Phenolic Profile of Nipa Palm Vinegar Determined by LC-MS

**Figure 1:**
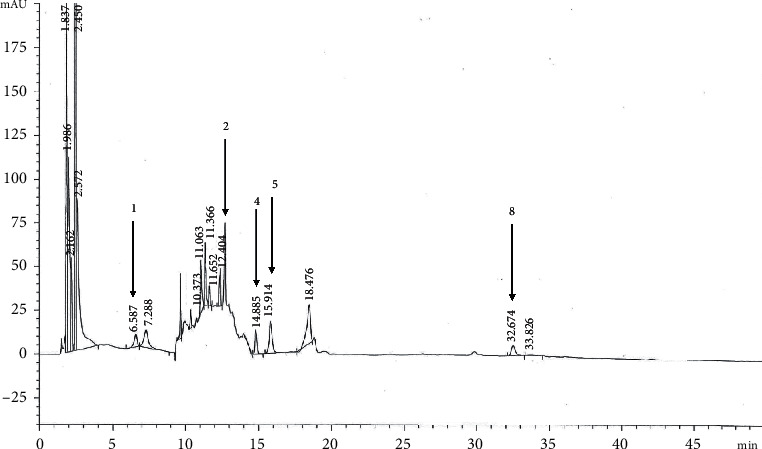
Chromatogram of phenolic compounds in nipa palm vinegar using LC-MS analysis.

LC-MS was used to identify of phenolic compounds. Gallic acid, tannic acid and hydroquinone, catechin, rutin, isoquercetin, eriodictyol, quercetin, apigenin, and kaempferol were used as standards (as shown in Table 1). The contents of gallic acid (peak 1), isoquercetin (peak 5), quercetin (peak 8), catechin (peak 2), and rutin (peak 4) were 14.14, 11.27, 10.33, 8.61, and 6.67 *µ*g/mL in NPV, respectively (as shown in Figure 1), while hydroquinone, tannic acid, eriodictyol, apigenin, and kaempferol were not detected in NPV.

### 3.3. Pancreatic Lipase Inhibitory Activity of Nipa Palm Vinegar

**Figure 2:**
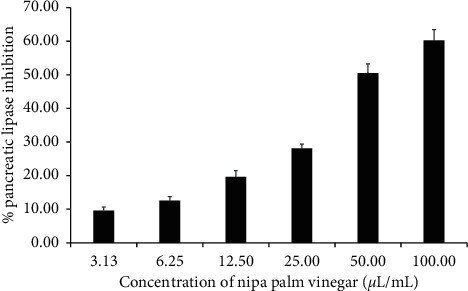
Pancreatic lipase inhibitory activity of nipa palm vinegar (NPV). The inhibitory activities of NPV against pancreatic lipase were determined at concentrations of 3.13, 6.25, 12.50, 25.00, 50.00, and 100.00 *µ*L/mL. Values are expressed as mean ± SD of triplicate measurements.

The inhibitory activity of nipa palm vinegar against pancreatic lipase is shown in Figure 2. The nipa palm vinegar inhibited pancreatic lipase activity by 9.55, 12.51, 19.65, 28.14, 50.53, and 60.22% at concentrations of 3.13, 6.25, 12.50, 25.00, 50.00, and 100.00 *µ*L/mL, respectively. The nipa palm vinegar had an IC_50_ value of 69.95 *µ*L/mL. Orlistat served as a pancreatic lipase inhibitor control, and a concentration of 2 *µ*g/mL reduced activity by 53.67%. These results show that the NPV showed a lipase inhibiting activity.

### 3.4. Pancreatic Cholesterol Esterase Inhibitory Activity of Nipa Palm Vinegar

**Figure 3:**
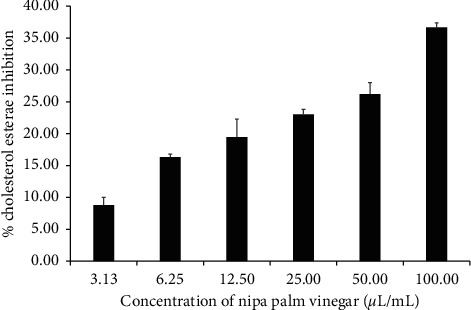
Pancreatic cholesterol esterase inhibitory activity of NPV. The inhibitory activities of NPV against pancreatic cholesterol esterase were measured at concentrations of 3.13, 6.25, 12.50, 25.00, 50.00, and 100.00 *µ*L/mL. Values are expressed as mean ± SD of triplicate measurements.

The nipa palm vinegar inhibited cholesterol esterase by 8.78, 16.33, 19.48, 23.02, 26.22, and 36.66% at concentrations of 3.13, 6.25, 12.50, 25.00, 50.00, and 100.00 *µ*L/mL, respectively (Figure 3). Simvastatin at a concentration of 300 *µ*g/mL was used as a positive control which inhibited cholesterol esterase by 41.66%. These findings show that the NPV has the ability to inhibit cholesterol esterase in a dose-dependent manner.

### 3.5. Effect of Nipa Palm Vinegar on Cholesterol Micellization

**Figure 4:**
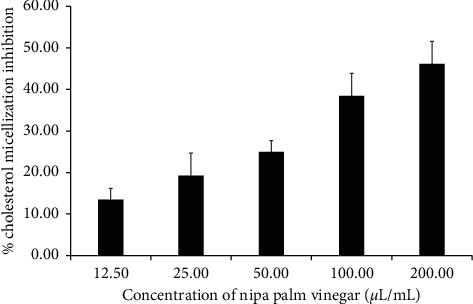
Effect of NPV on cholesterol micellization. The inhibitory activities of NPV against cholesterol micellization were measured at concentrations of 12.50, 25.00, 50.00, 100.00, and 200.00 *µ*L/mL. Values are expressed as mean ± SD of triplicate measurements.

The inhibition of cholesterol micellization by NPV at various concentrations is shown in Figure 4. The NPV suppressed cholesterol micellization by 13.46, 19.23, 25.00, 38.46, and 46.15% at concentrations of 12.50, 25.00, 50.00, 100.00, and 200.00 *µ*L/mL, respectively. Gallic acid at a concentration of 200 *µ*g/mL was used as a positive control which inhibited cholesterol micellization by 84.62%. These results show that the NPV can inhibit the formation of cholesterol micellization in a dose-dependent manner.

### 3.6. Correlation Analyses of Pancreatic Lipase Inhibition, Pancreatic Cholesterol Esterase Inhibition, and Cholesterol Micellization Inhibition of Nipa Palm Vinegar

**Figure 5:**
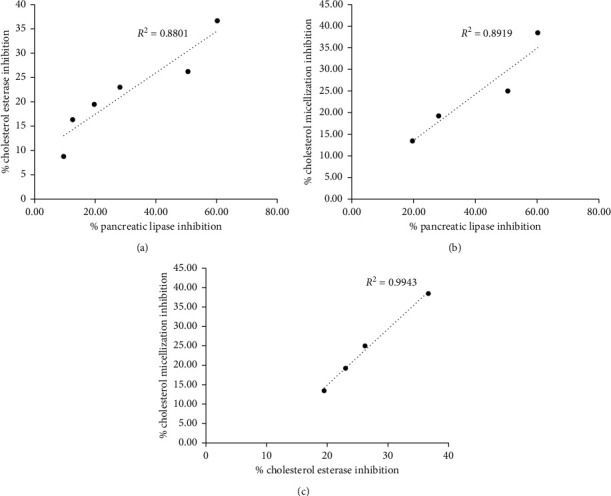
Correlation analyses. Values were determined for (a) % pancreatic lipase inhibition versus % cholesterol esterase inhibition, (b) % pancreatic lipase inhibition versus % cholesterol micellization inhibition, and (c) % cholesterol esterase inhibition versus % cholesterol micellization inhibition.

The linear regression analysis and correlation coefficients between the variables are presented in Figure 5. There were strongly positive correlations between % pancreatic lipase inhibition and % cholesterol esterase inhibition (*R*^2^ = 0.8801) at concentrations of 3.13, 6.25, 12.50, 25.00, 50.00, and 100.00 *µ*L/mL (Figure 5(a)). A strongly positive correlation was also found between the % pancreatic lipase inhibition and % cholesterol micellization inhibition (*R*^2^ = 0.8919) at concentrations of 12.50, 25.00, 50.00, and 100.00 *µ*L/mL (Figure 5(b)). Moreover, a positive correlation was also observed between % cholesterol micellization inhibition and % cholesterol (Figure 5(c)) esterase inhibition (*R*^2^ = 0.9943).

## 4. Discussion

Dyslipidemia is a key risk factor in the development of cardiovascular diseases which leads to increased morbidity and mortality [8]. Natural products have been used as an alternative medicine for the prevention and management of cardiovascular diseases [22]. There are many risk factors for dyslipidemia including genetic factors, hormonal abnormalities, and lifestyle factors. A diet especially high in fat is believed to be one of the greatest risk factors for the development of dyslipidemia [23]. Normally, pancreatic lipase is a key enzyme that hydrolyses ester linkages of triglyceride [24]. Therefore, pancreatic lipase inhibition is a goal to reduce fat absorption to control dyslipidemia. Pancreatic cholesterol esterase is an enzyme which hydrolyses cholesterol esters, so inhibition of pancreatic cholesterol esterase can reduce cholesterol absorption and may be useful for therapeutics for controlling cholesterol [25]. Moreover, the reduction of micelle formation is a target for lowering blood cholesterol level [21]. This study evaluated nipa palm vinegar for its total phenolic content and the presence of phenolic compounds as well as determined its ability to inhibit pancreatic lipase, inhibit cholesterol esterase, and inhibit cholesterol micellization.

Natural phenolic compounds as secondary metabolites of plants consist of phenolic acids, flavonoids, tannins, stilebenes, curcuminoids, coumarins, lignans, quinones, and others [26]. Phenolic compounds have been shown to have several pharmacological effects including antioxidant [27], anti-hyperlipidemic activity [28], anti-hypertensive activity [29], antimutagenic activity [30], anti-inflammatory activity [31], antidiabetic effect [32], and anticancer [33]. The total phenolic content in NPV was determined by the Folin–Ciocalteu assay. In this study, the NPV contained 167.10 ± 10.15 *µ*g GAE/mL. According to a previous study, nipa sap vinegar produced by surface fermentation contained a phenolic content of 253.98 ± 0.14 *µ*g GAE/mL and showed antioxidant activity against DPPH free radicals [34]. Moreover, phenolic compounds were determined by LC-MS, and the results showed that nipa palm vinegar contains gallic acid, isoquercetin, quercetin, catechin, and rutin.

Orlistat is a well-known pancreatic lipase inhibitor which is produced from *Streptomyces toxytricini*. It reacts with lipases at the active site serine by forming a covalent bond and thus inactivating the ability of these enzymes to hydrolyse dietary fat in the small intestine [35]. Adverse effects of orlistat are liquid stools, steatorrhea, fecal urgency, incontinence, flatulence, abdominal cramping, and fat-soluble vitamin deficiencies [36]. Recently, many researchers have been focused on the effects of natural products for the treatment of dyslipidemia [37]. Our study determined the inhibitory activity of NPV towards pancreatic lipase, and the results showed that NPV can inhibit pancreatic lipase in a concentration-dependent manner with an IC_50_ value of 69.95 *µ*L/mL. In a previous study, an aqueous extract of NPV was able to significantly reduce blood glucose in streptozotocin-induced diabetic rats [15]. The aqueous extract of NPV also stimulated insulin secretion in RIN-5F cells [16]. Moreover, nipa palm sap and its syrup inhibited *α*-glucosidase which hydrolyses polysaccharides [38].

Pancreatic cholesterol esterase exerts important functions in controlling the bioavailability of cholesterol from dietary cholesterol esters, contributing to the incorporation of cholesterol into mixed micelles, and in helping to transport free cholesterol to enterocytes [25]. It has previously been reported that inhibition of pancreatic cholesterol esterase and the solubility of cholesterol micelles by gallic acid, catechin, and epicatechin from grape seed extract results in delaying the absorption of cholesterol [20]. The current findings revealed that NPV inhibited pancreatic cholesterol esterase and the solubility of cholesterol micelles in a concentration-dependent manner. There was a positive correlation between pancreatic lipase inhibition and pancreatic cholesterol esterase inhibition. Moreover, cholesterol micellization inhibition had a positive correlation with pancreatic lipase inhibition and pancreatic cholesterol esterase inhibition. Therefore, it could be hypothesized that the NPV can inhibit pancreatic cholesterol esterase and may protect against cholesterol micellization.

There are several reports demonstrating anti-dyslipidemia activity of natural compounds. Gallic acid has been reported to decrease levels of serum triglycerides, total cholesterol, and low-density lipoprotein in high-fat induced dyslipidemia in rats [39]. Similarly, quercetin had a strong inhibitory activity on pancreatic lipase [40], and it also reduced serum levels of triglycerides and cholesterol in a rabbit model of high-fat diet-induced atherosclerosis [41]. Moreover, isoquercetin, a glucoside derivative of quercetin, improved hepatic lipid accumulation through activating the AMPK pathway and inhibiting TGF-*β* signalling in a high-fat diet-induced nonalcoholic fatty liver disease rat model [42]. A previous study showed that green tea catechin suppressed cholesterol absorption in the small intestine and reduced serum cholesterol concentrations [43]. Other studies have also demonstrated the antiobesity effects of rutin by decreasing serum lipid profiles and leptin [44].

According to our *in vitro* finding, it can be hypothesized that phenolic compounds including gallic acid, isoquercetin, quercetin, catechin, and rutin from NPV may be the main active compounds with possible cholesterol-lowering effects through inhibition of pancreatic lipase and cholesterol esterase activities as well as the inhibition of solubility of cholesterol micelles. More *in vivo* studies in animal and clinical human studies are required to determine the anti-dyslipidemia activity of NPV and to confirm its mechanism for the aim of application in the prevention and treatment of dyslipidemia.

## 5. Conclusion

Our results indicate that nipa palm vinegar may delay the digestion of a high-fat diet and absorption through small intestine through mechanisms such as the inhibition of pancreatic lipase and cholesterol esterase activities as well as through the inhibition of solubility of cholesterol micelles. These results indicate that NPV could be used as a natural source of bioactive compounds with antilipidemic activity. Nevertheless, NPV should be extensively evaluated by animal and clinical human studies.

## Figures and Tables

**Table 1:** LC-MS characteristics of phenolic compounds in nipa palm vinegar.

Peak	Compounds	Retention time (min)	[M + H]^+^ (m/z)	Contents (*µ*g/mL)
1	Gallic acid	5.60 ± 0.99	188.0	14.14 ± 0.07
2	Catechin	12.40 ± 0.08	185.0	8.61 ± 0.32
3	Tannic acid	12.82 ± 0.04	503.0	ND
4	Rutin	15.05 ± 0.17	649.0	6.67 ± 0.03
5	Isoquercetin	16.12 ± 0.21	329.0	11.27 ± 0.12
6	Hydroquinone	24.56 ± 0.20	289.0	ND
7	Eriodictyol	30.71 ± 0.55	327.0	ND
8	Quercetin	33.02 ± 0.36	341.0	10.33 ± 0.16
9	Apigenin	41.08 ± 0.40	271.0	ND
10	Kaempferol	42.37 ± 7.97	287.0	ND

ND: not detected; data are expressed as mean ± standard deviation in triplicate.

## Data Availability

The data used to support the findings of this study are available from the corresponding author upon request.
